# Supplementary material for: Short-Term Outcomes of Conventional Laparoscopic versus Robot-Assisted Distal Pancreatectomy for Malignancy: Evidence from US National Inpatient Sample, 2005–2018
Source: Cancers (Basel). 2024 Feb 29;16(5):1003. doi: 10.3390/cancers16051003 (PMC10930702; doi:10.3390/cancers16051003)
Supplement: Supplementary file 1 [file cancers-16-01003-s001.zip › cancers-2850137-supplementary.pdf]

Supplementary

Table S1. ICD codes used in the study

	ICD-9 code	ICD-10 code
Malignant neoplasm of pancreas	**CM:** 157	**CM:** C25
Conventional laparoscopic distal pancreatectomy (DP)	**PCS:** 52.52 AND 54.21	**PCS:** 0FBG3ZZ, 0FBG4ZZ, 0FBG8ZZ, 0FTG4ZZ.
Robot-assisted laparoscopic distal pancreatectomy (DP)	**PCS:** 52.52 AND 17.4	**PCS:** 0FBG3ZZ, 0FBG4ZZ, 0FBG8ZZ, 0FTG4ZZ AND 8E0W0CZ, 8E0W3CZ, 8E0W4CZ, 8E0W7CZ, 8E0W8CZ, 8E0WXCZ
malignant neoplasm of head of pancreas	**CM:** 157.0	**CM:** C25.0
Proximal Pancreatectomy (Whipple procedure)	**PCS:** 52.51, 52.7	**PCS:** 0DT9*, 0DB9*
AMI	**CM:** 410 **DXCCS:** 100	**CM:** I21 DXCCSR_CIR009>0
CVA	**CM:** 433.01, 433.10, 433.11, 433.21, 433.31, 433.81, 433.91, 434.00, 434.01, 434.11, 434.91, 436, 430, 431 **DXCCS:** 109	**CM:** I60, I61, I63, I69 DXCCSR_CIR020>0
VTE	**CM:** 415, 451-453, 671, 673, 997.2	**CM:** I26.0, I26.9, I80.1-I80.3, I80.8, I80.9, I82.0-I82.3, I82.8, I82.9, O08.2, O22.3, O87.1, O88.2, I81, I82
Periprocedural shock, hypertension, or other cardiovascular complications	**CM:** 998.00, V58.89, 909.3, 997.91, 997.1, 997.2, 997.7	**CM:** T81.1, I97.3, I97.88, T81.7
Pneumonia	**CM:** 486, 481, 482.8, 482.3	**CM:** A48.1, J12 J13, J14, J15, J16, J17, J18, J69.0, J95.4, J95.851
Postprocedural pneumothorax	**CM:** 512.1	**CM:** J95.811
Postprocedural air leak	**CM:** 512.2	**CM:** J95.812
Acute respiratory failure	**CM:** 518.5, 518.81-518.84	**CM:** J95.1, J95.82
Pulmonary collapse (atelectasis)	**CM:** 518	**CM:** J98.11
Sepsis	**CM:** 995.9, 996.64, 038, 999.3, 790.7, 041.x, 785.52	**CM:** R78.81, A41, R65.2, T81.4, T80.2, A42.7, A22.7, B37.7, A26.7, A28.2, A54.86, B00.7, A32.7, A24.1, A39.2, A20.7, A21.7, A48.3
Infection	**CM:** 001-139	**CM:** L00-L08, B99, T81.43, O86.03
Mechanical ventilation	**PCS:** 96.7	**PCS:** 5A1935Z, 5A1945Z, 5A1955Z
Postoperative blood transfusion	**PCS:** 99.04	**PCS:** 30233N1

Other complications of respiratory system	**CM:** 997.32, 997.39, 519.0	**CM:** J95.88, J95.89, J95.0, J98.0
Perforations of organ or vessels	**CM:** 998.2	**CM:** D78.12, G97.49, E36.12, K91.71, N99.72, T88.8XXA, M96.821, L76.12
Smoking	**CM:** 305.1, V15.82, 989.84	**CM:** Z71.6, Z72.0, Z86.43, Z87.891, F17, O99.33, T65.2
Spleen removed	**PCS:** 41.43, 41.5	**PCS:** 07TP*, 07BP*
Coronary artery disease	**CM:** 411-414	**CM:** I25
Congestive heart failure	CM_CHF	**CM:** I09.9, I11.0, I13.0, I13.2, I25.5, I42.0, I42.5-I42.9, I43.x, I50.x, P29.0
Persistent anemia	**CM:** 285	**CM:** D63, D64
Diabetes	CM_DM, CM_DMCX	**CM:** E10-E14
Hypertension	**CM:** 401–405 CM_HTN_C	**CM:** I10
Cerebrovascular disease	**CM:** 362.34, 430.x-438.x	**CM:** G45.x, G46.x, H34.0, I60.x-I69.x
Chronic pulmonary disease	**CM:** 416.8, 416.9, 490.x-505.x, 506.4, 508.1, 508.8	**CM:** I27.8, I27.9, J40.x-J47.x, J60.x-J67.x, J68.4, J70.1, J70.3
Hyperlipidemia	**CM:** 272.0, 272.1, 272.2, 272.3, 272.4, 272.5, 272.8, 272.9	**CM:** E78.0~E78.9
Drug abuse	CM_DRUG	**CM:** Z71.5, F10-F19
Severe liver disease	**CM:** 456.0–456.2, 572.2–572.8	**CM:** I85.0, I85.9, I86.4, I98.2, K70.4, K71.1, K72.1, K72.9, K76.5, K76.6, K76.7
Moderate or severe renal disease	**CM:** 403.01, 403.11, 403.91, 404.02, 404.03, 404.12, 404.13, 404.92, 404.93, 582.x, 583.0-583.7, 585.x, 586.x, 588.0, V42.0, V45.1, V56.x	**CM:** I12.0, I13.1, N03.2-N03.7, N05.2-N05.7, N18.x, N19.x, N25.0, Z49.0-Z49.2, Z94.0, Z99.2
Rheumatic disease	**CM:** 446.5, 710.0-710.4, 714.0-714.2, 714.8, 725.x CM_ARTH	**CM:** M05.x, M06.x, M31.5, M32.x-M34.x, M35.1, M35.3, M36.0

ICD, International Classification of Diseases; CM, Clinical Modification; PCS, procedure code; DP, distal pancreatectomy; AMI, acute myocardial infarction; CVA, cerebral vascular accident; VTE, venous thromboembolism.
